# *eCerto*—versatile software for interlaboratory data evaluation and documentation during reference material production

**DOI:** 10.1007/s00216-023-05099-3

**Published:** 2023-12-18

**Authors:** Jan Lisec, Sebastian Recknagel, Carsten Prinz, Kristin Vogel, Matthias Koch, Roland Becker

**Affiliations:** 1 Department of Analytical Chemistry and Reference Materials, Bundesanstalt für Materialforschung und -prüfung (BAM), Berlin, Germany; 2 Section eScience (S.3), Bundesanstalt für Materialforschung und -prüfung (BAM), Berlin, Germany

**Keywords:** Reference material, Statistics, Software, Collaborative trial

## Abstract

The statistical tool *eCerto* was developed for the evaluation of measurement data to assign property values and associated uncertainties of reference materials. The analysis is based on collaborative studies of expert laboratories and was implemented using the R software environment. Emphasis was put on comparability of *eCerto* with SoftCRM, a statistical tool based on the certification strategy of the former Community Bureau of Reference. Additionally, special attention was directed towards easy usability from data collection through processing, archiving, and reporting. While the effects of outlier removal can be flexibly explored, *eCerto* always retains the original data set and any manipulation such as outlier removal is (graphically and tabularly) documented adequately in the report. As a major reference materials producer, the Bundesanstalt für Materialforschung und -prüfung (BAM) developed and will maintain a tool to meet the needs of modern data processing, documentation requirements, and emerging fields of RM activity. The main features of *eCerto* are discussed using previously certified reference materials.

**Graphical Abstract:**

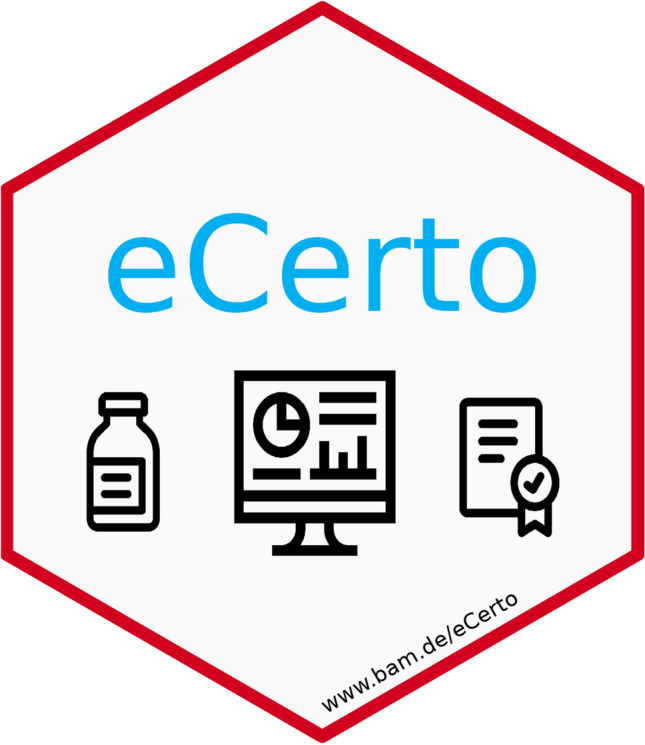

**Supplementary Information:**

The online version contains supplementary material available at 10.1007/s00216-023-05099-3.

## Introduction

Reference materials (RM) are prominent tools in quality control of chemical content analyses. Applications include instrument calibration with RMs displaying no matrix influence on the analytes’ detector response and the quality control of complete analytical procedures via matrix reference materials. Especially in the latter case, the property values and associated uncertainties are often assigned using interlaboratory comparisons involving expert laboratories. A respective program, SoftCRM, has been developed for the evaluation of certified values and uncertainties on basis of the Community Bureau of Reference (BCR) guidelines [1] about 25 years ago [2]. It includes procedures for the assessment of homogeneity and stability that may contribute to the property values, uncertainties and tests for variances, normality, and outlying observations. However, an updated version is not widely accessible and modern requirements for graphical representation of results and textual output demand a customized program for facile data processing, documentation, and transfer to data sheets, certificates, and reports. BAM is an accredited provider of reference materials in many areas of chemical analyses [3] and ventures increasingly on RM-related activities. This requires a tool that enables easy and reliable data import, data evaluation, and reporting. This tool should allow further development with the evolving scope of matrices and parameters. Based on the procedures implemented in SoftCRM [1] and considering the currently valid versions of ISO 17034 [4] and ISO Guide 35 [5], *eCerto* was developed using the R environment and enables the analyst responsible for a given RM project to handle and interactively explore homogeneity, stability, and intercomparison data. It also allows for evaluating assigned values and uncertainties along with up-to-date documentation and reporting.

## Technical specification

### Software environment

The open-source programming environment R was used to develop *eCerto* because of the availability of a plethora of readily implemented statistical tests. All incorporated tests are described within the documentation of the tool and links to function documentation of the employed packages are provided. Some tests were re-implemented to extend functionality (i.e., to allow sample numbers of *n*>30 for the Cochran test) or newly implemented if no package was available (i.e., Arrhenius approach to investigate parameter stability). Most R packages provide a collection of functions that can be used on the command line. However, to allow convenient data processing also for users without programming knowledge, we implemented a graphical user interface using the R package *Shiny* allowing to start *eCerto* as an app.

Shiny apps are HTML/JS based and can run in any browser with access to an R session in the background. For testing purposes or simply for convenience, we provide a dedicated server which allows running *eCerto* without any installation (www.bam.de/eCerto). This server is maintained by BAM without any limitations. It will be kept available in the foreseeable future to ensure access to, as well as traceability of, data sets processed in house. Users without internet access can run *eCerto* locally after installing R and the *eCerto* package. This could also be reasonable for especially sensitive data and further ensures long-term availability of the software.

The source code of *eCerto* is accessible on GitHub (https://github.com/janlisec/eCerto), where also bug reports and user requests can be filed. The functionality of the app (and where appropriate the code) is well documented and modularly structured, which allows extending *eCerto* if necessary. To ensure functional integrity of the software after prospective updates, automatic testing was implemented using the *testthat* package and establishing approximately 200 tests and 80% code coverage.

### Software structure

**Fig. 1:**
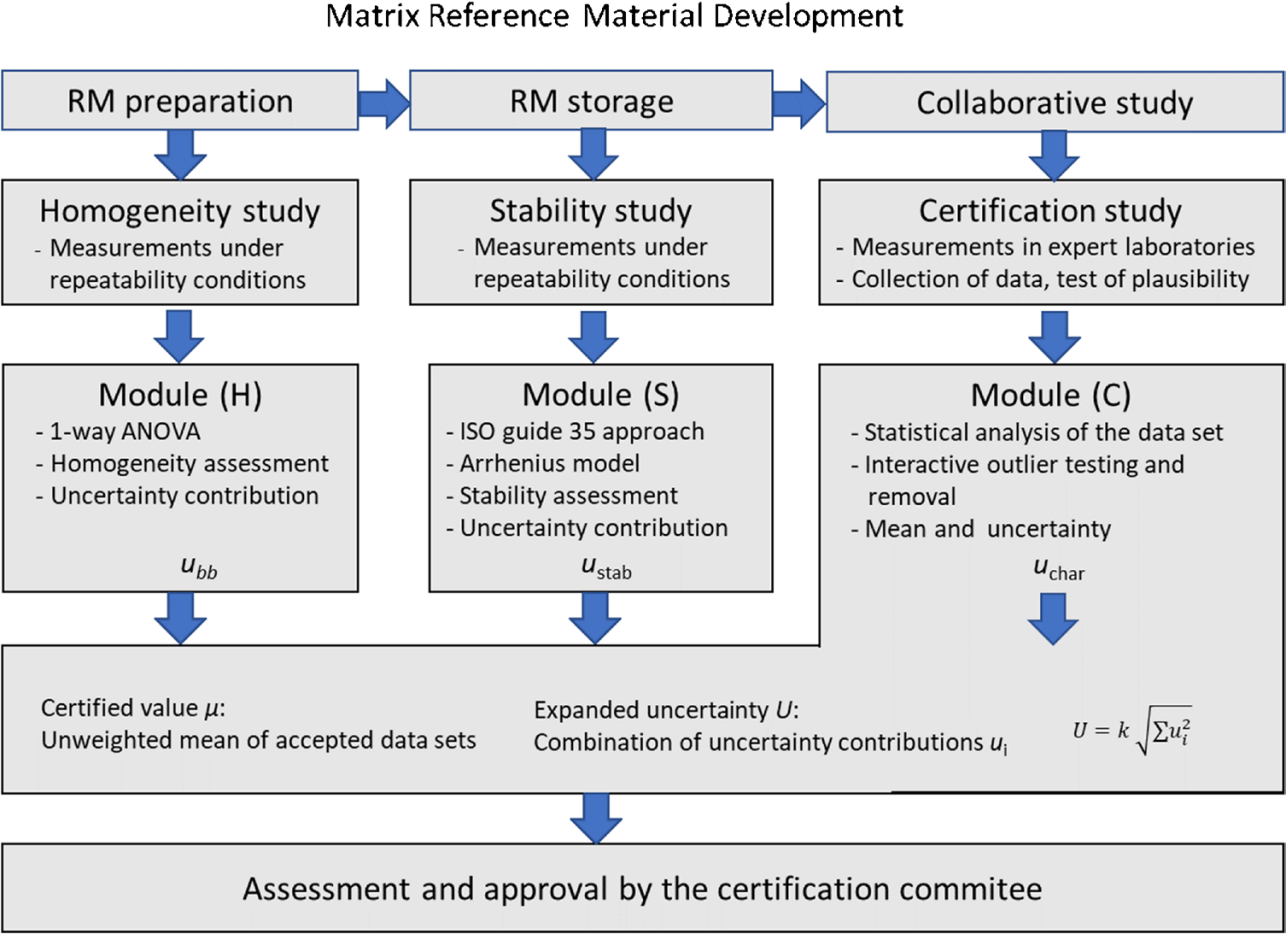
*eCerto* modules (H), (S), and (C) as part of reference material development. The whole process is fully controlled by the certification committee from initial planning to final approval and certification

The user interface of *eCerto* is composed of five modules or sub-pages following the workflow of a certification process (Fig. 1). Generally, after preparation and bottling of the material, the results of a homogeneity study need to be evaluated and the homogeneity (H) of all relevant parameters across all units of the produced batch needs to be assessed. Further, in cases where parameters cannot be considered as intrinsically stable, the stability (S) of the certified properties needs to be evaluated. A collaborative study (C) with selected expert laboratories is conducted to determine a reasonable estimate of value and uncertainty for each property. Statistical tests and graphical output for each of these tasks are provided in the corresponding modules H, S, and C of *eCerto*. The statistical tests and functionality of the individual modules are detailed in the following sections using actual data sets of previously published CRMs.

Additionally, *eCerto* contains a start module, which provides centralized data upload functionality and backup options. Data upload is currently based on Excel files with straightforward layout and examples provided in the tool. Additional data import formats can be incorporated in the respective module of *eCerto*, either by extending the code in the Git repository through experienced users or upon e-mail request to the corresponding author. The decision to use Excel was made as it is convenient for most users. Excel files can be easily locked for editing once the data is collected, ensuring traceability of the original data, and preventing data manipulation, especially as *eCerto* imports and stores data including the source file names. While homogeneity and stability are each accessed in one, often the same, laboratory, data from collaborative studies are often collected from Excel files distributed to the selected expert laboratories. Here, *eCerto* allows importing all individual files in parallel, eliminating the need to combine participant data upfront which is tedious and implies a risk of copy/paste errors. All imported data and user options can be stored in a combined file (RData format) for traceability and can be re-imported to *eCerto* when needed.

Besides a module for post certification monitoring, which allows estimating and monitoring stability during the period of sale of the CRM, *eCerto* contains an extensive documentation section which comprises descriptions of all methods, statistical tests, table column definitions, figure contents, and user options. To improve usability, each help section can also be accessed via hyperlinks near the respective part of the software described in this help section. To facilitate recalculations, applied formulae are provided in help sections. Therefore, formula depiction will be kept to a minimum in this manuscript.

### Data evaluation

#### Homogeneity study

**Fig. 2:**
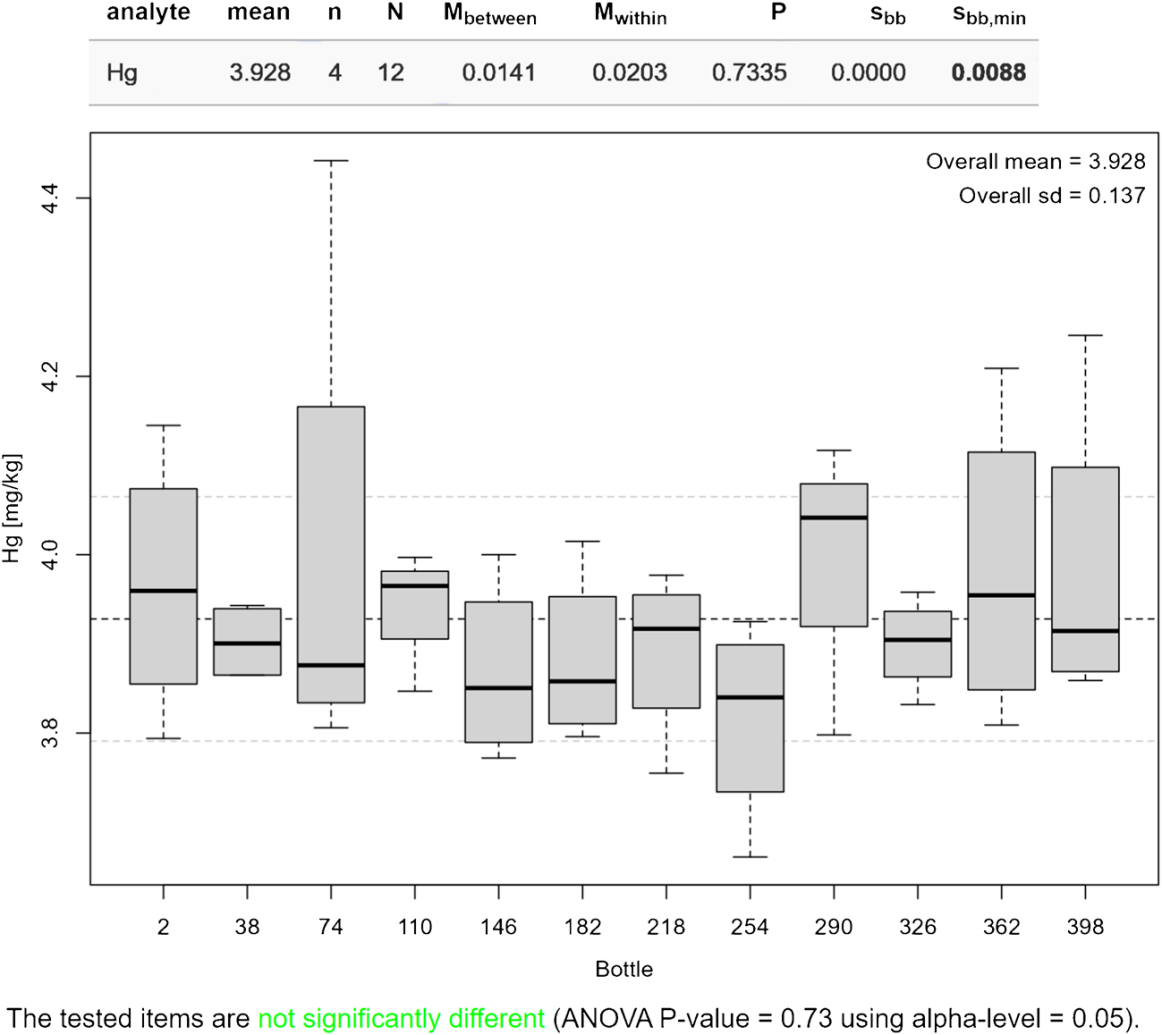
Screenshot of *eCerto* Fig. H1 and Tab. H1 showing the box plot representation of the homogeneity study for the parameter Hg of certified reference material BAM-U115. The relevant statistical parameters are displayed as annotations to the figure and are additionally tabulated

ISO Guide 35 [5] suggests several designs to investigate the homogeneity of a reference material. Currently, the homogeneity module (H) of *eCerto* offers evaluation of data obtained from the so-called nested design where the parameter content is determined in *N* bottles from the batch for *n* times in a randomized order under repeatability conditions. Figure 2 depicts the results obtained from 12 bottles of certified soil reference material BAM-U115 [6] analyzed four times each under repeatability conditions for the content of mercury. The Excel input file with the homogeneity measurement data is provided as SI 1.

Figure 2 shows the evaluation based on 1-way ANOVA yielding the mean square errors between and within the bottled units (*M*_between_, *M*_within_) in relative terms. The contribution to the uncertainty of the property value is obtained as relative standard deviation between the bottled units *s*_bb_ of the batch under investigation using the procedure laid down in ISO Guide 35 [5]. This guide sets *s*_bb_ = 0 in cases where *M*_within_ > *M*_between_ and suggests calculating *s*_bb,min_, the standard deviation of maximal between bottle variation that can be masked by within bottle variation, instead. Both values are given in relative terms in table H1 (Fig. 2). The help function of *eCerto* provides details regarding the formulae used for the calculations. *eCerto* does not restrict the number of bottles *N* and replicate *n* analyses but it is expected that the user follows the recommendations of ISO Guide 35 and the certification committee (Fig. 1) for the homogeneity study.

#### Stability study

To ensure that a parameter in a prospective RM is stable over time, replicate measurements of RM units kept under storage conditions are performed over a period (usually 12 months). If the slope of a linear model of this data is not significantly different from 0, this indicates that the property is stable. Nevertheless, an uncertainty contribution* u*_stab_ = |*t*_*cert*_ x *s(b*_*1*_*)*| can be calculated where *s(b*_*1*_*)* is the standard error of the slope and *t*_*cert*_ is the planned lifetime of the material. *eCerto* allows to upload isochronally measured time course data and will compute a linear model, the according slope, *p*-value, and *u*_stab_ for each parameter. The user can decide to transfer *u*_stab_ to the C module to incorporate this uncertainty term in the certificate.

ISO Guide 35 [5] describes the option to perform accelerated stability studies for specific exposure conditions. To this end, the procedure to evaluate data from sample time series stored at different temperatures was implemented in *eCerto*. Evaluating such data allows testing for potential degradation processes of the parameters and ultimately to infer the optimal storage conditions for a RM. This approach has been used for RM production at BAM for over 20 years and was originally described in Bremser et al. [7]. Basically, the investigation of RM samples stored at different temperature levels and time periods up to 12 months, allows calculating a linear model at each investigated temperature yielding temperature-dependent reaction rates *k*_eff_(*T*). These reaction rates can be combined in an Arrhenius approach to infer degradation rate, and inversely estimate stability, at any temperature covered by the study layout.

To test if *eCerto* faithfully reproduces previous results obtained by this approach, we reanalyzed data published in Riedel et al. [8]. The time axis of the original data describing the storage time under stress conditions, i.e., at temperature levels higher than the reference temperature of −80 °C, was published in units of month. Because stability measurements are often continued during post certification monitoring, the data upload in *eCerto* expects a date value (day of measurement). Therefore, time values had to be re-encoded by setting the reference samples (stored exclusively at −80 °C) to a fictive date (2022-01-01) and adjusting all other samples accordingly (0.5-month samples were specified as 2022-01-15, 1-month samples as 2022-02-01, etc.). The resulting Excel file is provided as SI 2. Loading it in *eCerto* instantly leads to the calculation of all reaction rates *k*_eff_(*T*) and combines these in the Arrhenius plot. Providing the certified value *µ*_C_ and expanded uncertainty *U*_abs_ retrieved from the corresponding collaborative study manually (as this data is not contained in the stability data file), *eCerto* provides identical expiry times compared to Tab. 4 of the original publication (Fig. 3).

**﻿Fig. 3:**
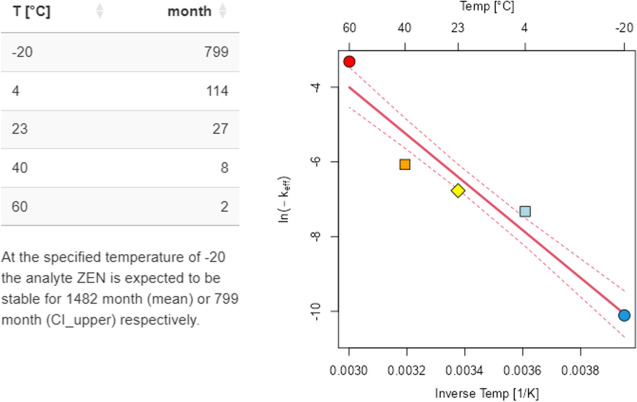
Partial screenshot of the S module (Arrhenius) from *eCerto* re-evaluating data from CRM ERM-BC715. The main results (Arrhenius model and resulting expiry times at specific temperature levels) are identical to the previously published values. Symbol shape and background in the figure indicate temperature level. Red dotted lines represent the CI of the regression line of the linear model. The upper CI is used to calculate prospective shelf life as shown in the table

The original data were evaluated in a complex Excel spreadsheet which was confusing and susceptible to unintentional misentry due to its size and the numerous interim calculations connected by cell references. In *eCerto*, calculation steps are fixed, and focus is put on the results. Intermediate results are depicted for reference in a compact layout and relevant formulas can be shown by clicking on the help links atop of each figure or table.

#### Certification study

After the results of the certification analyses have been received from the expert laboratories, they are evaluated using the certification module (C) of *eCerto.* The original dataset of each parameter is tested for outlying and straggling results representing levels of significance of 1% and 5%, respectively. Following the outline of SoftCRM, this comprises several tests of normality (QQ-plot, KS test, skewness, and kurtosis), tests of variance homogeneity (Bartlett test) and compatibility of laboratory data (Scheffé test), and tests of deviating laboratory means (single and double Grubbs tests, Dixon test) and deviating laboratory variances (Cochran test). The critical values, including tables, formulas, and original sources, are provided in detail in the online documentation (section “Statistical test details”).

In contrast to SoftCRM, the outlier test named after Nalimov [9, 10] is not included in *eCerto*. As Streuli already pointed out [11], the critical values taken from Nalimov [12] were not meant for this purpose. This error led to the “Nalimov test” as a test for outlying observations being allegedly stricter than the Grubbs test. However, using the correct values listed by Nalimov [12] which were reprinted from Grubbs (1950) [13], the procedure mentioned by Nalimov represents a version of Grubbs’ test as already implemented in *eCerto* and suggested by ISO 5725 with the test statistics from Grubbs’ paper published in 1972 [14].

**Fig. 4:**
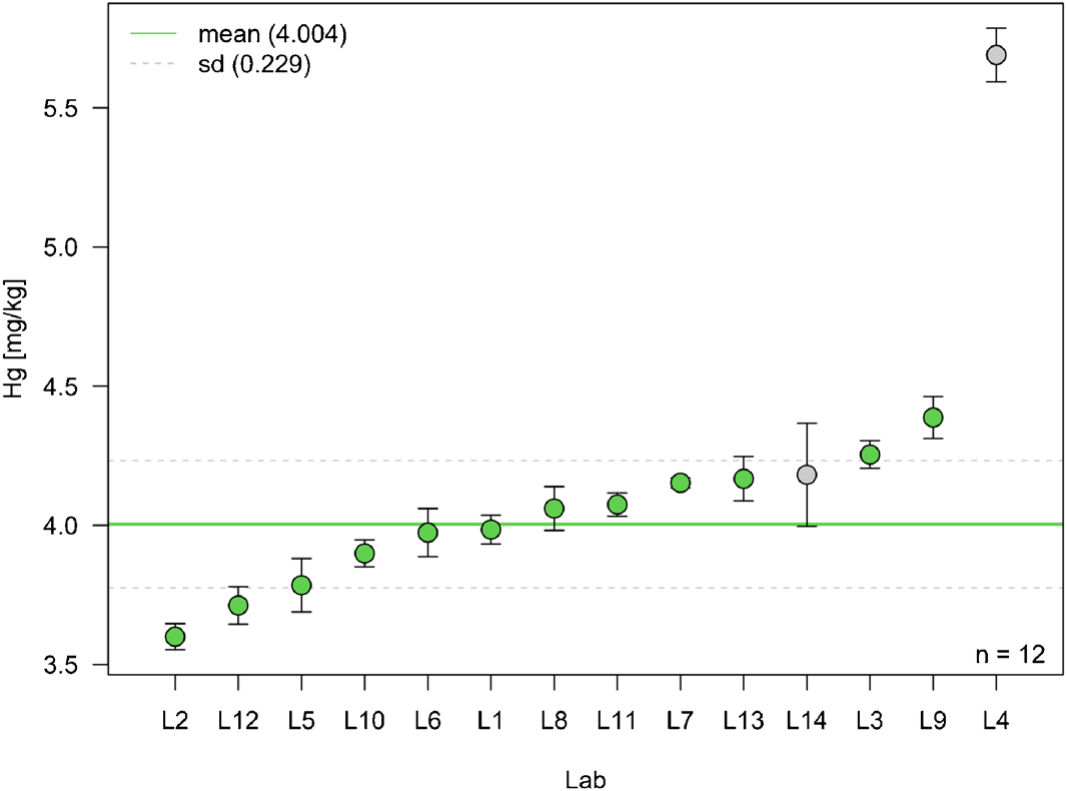
Screenshot of *eCerto* Fig. C1 showing the distribution of all measurement values for the parameter Hg from soil CRM BAM-U115. This figure allows a visual control of the lab mean distribution, excluded outliers (gray symbols), and prospective certified values (see plot legend)

**Fig. 5:**
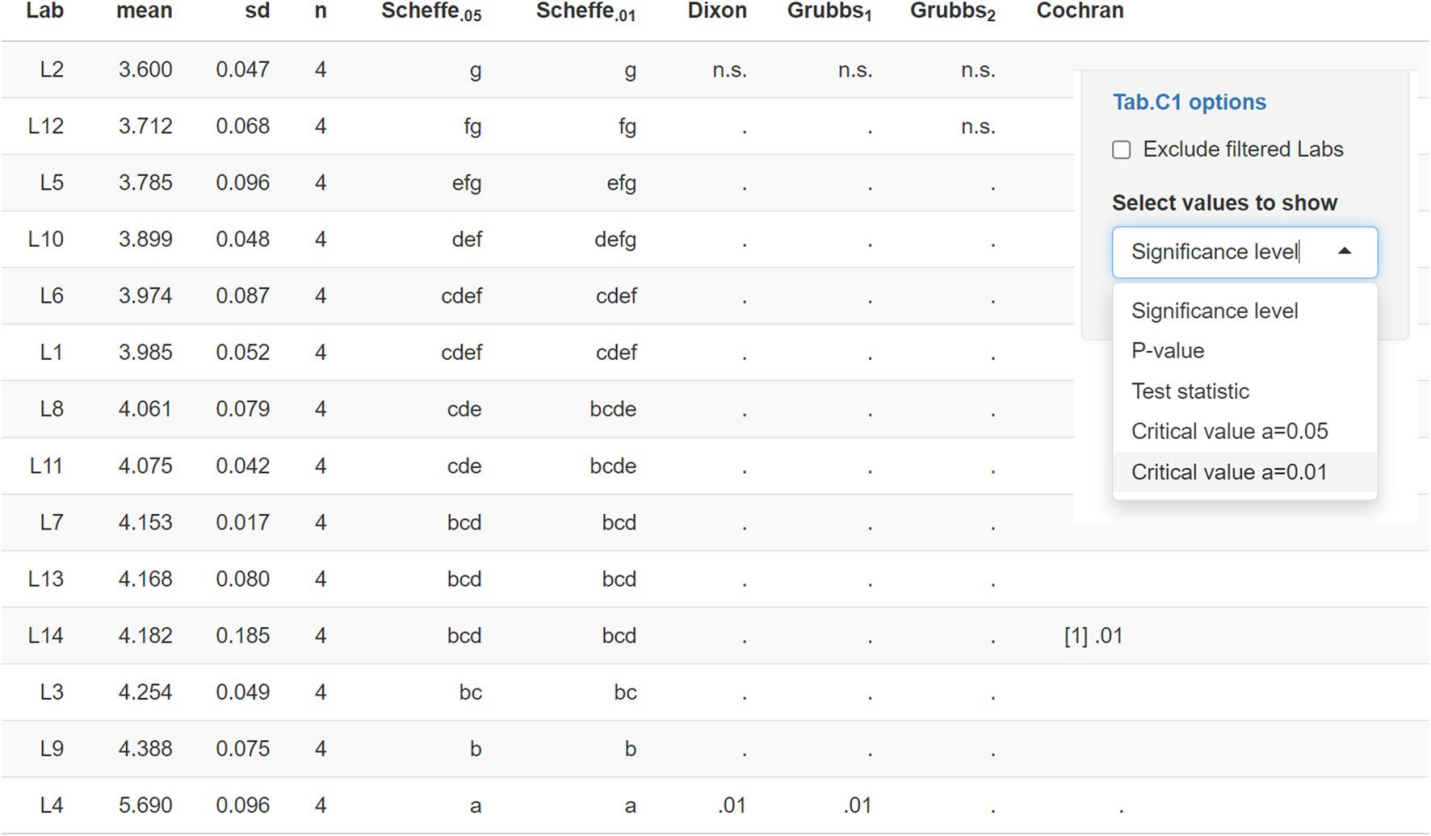
Screenshot of *eCerto* Tab.C1 showing the results of applied statistical tests. The table is automatically updated upon different user interactions (i.e., outlier removal) and can show different values per test as indicated on the right-hand side

To exemplify the performance of *eCerto*, data used to certify the content of Hg in soil reference material BAM-U115 [6] were reevaluated. The Excel input files and the RData output file are provided as SI 3 and SI 4. As may be anticipated from the distribution of all measurement values sorted according to lab mean (Fig. 4), lab L4 is suspected of being an outlier. To confirm this, *eCerto* presents optionally either test statistics,* p*-values, critical values, or significance levels of the various outlier tests in a compact tabular format (Fig. 5). Besides lab L4 (deviating mean, Grubbs test), also lab L14 is found to be an outlier at *α*=0.01 (deviating variance, Cochran test).

The critical values of all performed tests were in accordance with previous analyses. Also, the process of consecutive removal of two labs (L4 and L14) was identical, leading to similar property values. However, the evaluation with *eCerto* was much faster compared to SoftCRM, leading to the result in a modern output format in just a few clicks. It should be noted that outlier detection and subsequent removal should not be fully automatized but based on the decision of the user. This may account for the evaluation of technical reasons for outlying observations.

We provide as SI 5 input data of another interesting example which was used to compare *eCerto* output with previous results. Specifically, this data, containing values from the parameters Ca and Mg of ERM-ED103, demonstrates the requirement to remove suspected outliers consecutively because for both chemical elements two labs with extreme values are visible in the plot. However, in both cases, one of the two outliers becomes statistically significant only after the most extreme value was removed from the analysis. The SI file can be directly opened in the online version of *eCerto*.

#### Property values and expanded uncertainties

**Fig. 6:**
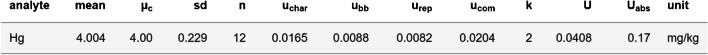
Screenshot of *eCerto* Tab. C3 with certified value *µ*_c_, individual uncertainty contributions *u*, and expanded *U*_abs_

Figure 6 shows the *eCerto* table C3 leading to the property value for the Hg content and its expanded uncertainty in BAM-U115. As outlined in Fig. 1, the certified value *µ*_c_ is the unweighted mean of laboratory means obtained in module (C). While the uncertainty from the interlaboratory comparison *u*_char_ is automatically transferred to Tab. C3, the uncertainty between the bottles *u*_bb_, which is equivalent to the greater of both values *s*_bb_ and *s*_bb,min_ (Fig. 2), is added by the user with the transfer option in the homogeneity module (H). Further uncertainty contributions may be added by the user. In case of this particular CRM, no uncertainty contribution due to stability was considered but the operator added an additional uncertainty factor *u*_rep_ covering the precision of laboratory means of the collaborative study participants [6]. Tab. C3 (Fig. 6) contains the relative uncertainty contributions *u*_char_,* u*_bb_, and* u*_rep_ and the relative combined uncertainty *u*_com_. The expanded relative uncertainty *U* is obtained after choosing the expansion factor* k*; the default value in *eCerto* is *k*=2. In *eCerto*, the user may round *µ*_c_ and *U*_abs_ as suggested in the certification panel and according to a procedure laid down in DIN 1333 [15]. The certified value *µ*_c_ for Hg in BAM-U115 of 4.00 ± 0.17 mg/kg obtained by *eCerto* is identical to the originally certified values calculated using SoftCRM [6].

#### Post certification monitoring

Post certification monitoring (PCM) is the process to ensure that certified values of RMs remain stable within the certified interval. Two options for PCM are available in *eCerto*.

**Fig. 7:**
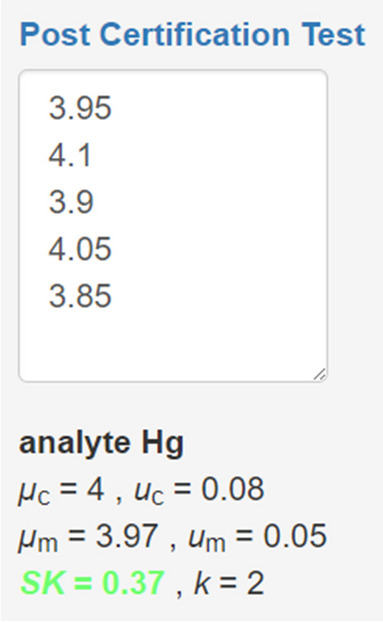
Simple input to allow PCM by calculation of a stability criterion *SK*. Measured values can be entered manually or copy/pasted from a spreadsheet. *SK* is colored red when its value exceeds the expansion factor* k* indicating potential instability of the material. Formula and variable definitions can easily be accessed by the user using the help link above the input field

In a simple approach, the user can load the *eCerto* backup file created during the certification process, select a parameter, and enter some measurement values obtained from replicate measurements of a RM unit (Fig. 7). Mean and standard uncertainty of these values will be compared with the certified values to compute a stability criterium $SK=\left|\mu_c-\mu_m\right|/\sqrt{\mu_c^2+\mu_m^2}$ where *µ*_m_ is the mean and *u*_m_ is the standard uncertainty of PCM measurements, and where *u*_c_ = *u*_com_ x *µ*_c_ is the absolute uncertainty without expansion. If *SK* is smaller than the expansion factor *k* selected in the certification process, this indicates that the CRM is stable for this parameter.

However, this approach does not account for a potential deviation of analytical values the lab conducting the PCM might have compared to the certified value. To give an extreme example, if the PCM lab measured the parameter of interest at the lower end of the distribution in the collaborative study, PCM might wrongly lead to a *SK* indicative of a rejection. Also, the simple test of *SK* does not allow to infer a prospective shelf life. Both issues can be addressed using a long-term stability (LTS) approach described in the following.

The basis of the long-term monitoring is a linear regression, y = *b*_0_ + *b*_1_ *x*, where *y* represents PCM measurements regressed on the time since certification in month (*x*) and yielding a slope of the regression line (*b*_1_).

To estimate the LTS prospectively, measurement data are continuously collected and stored. Similar to the other modules of *eCerto*, initially data needs to be prepared in a specific Excel format, providing measurement and metadata. After import, the metadata information cannot be edited further in *eCerto* to prevent unintentional modifications. Therefore, the user should carefully check the data provided. For example, the uncertainty definition, which could be 1*s* or 2*s* (to indicate single or double variance), *CI* (confidence interval), or 1*s*_*x*_ or 2*s*_*x*_ (to indicate the relative single or double variance), should match the provided uncertainty value as this will affect downstream calculations.

**Fig. 8:**
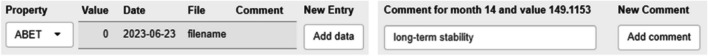
Screenshot of input forms to enter new data points (left) and individual comments (right). Only comments can be modified at any time, while data points are protected against retrospective modification

After data upload, the user can conveniently add new data points for a selected property (Fig. 8). Comments on a data point are optional. To prevent unintentional modification of the measurement data, only the comments can be changed by the user. Changing comments is conveniently done by selecting a data point, either in Tab. L1 or in Fig. L1 in *eCerto*, entering a new text in the comment input field (Fig. 8) and confirming the change by clicking the respective button

**Fig. 9:**
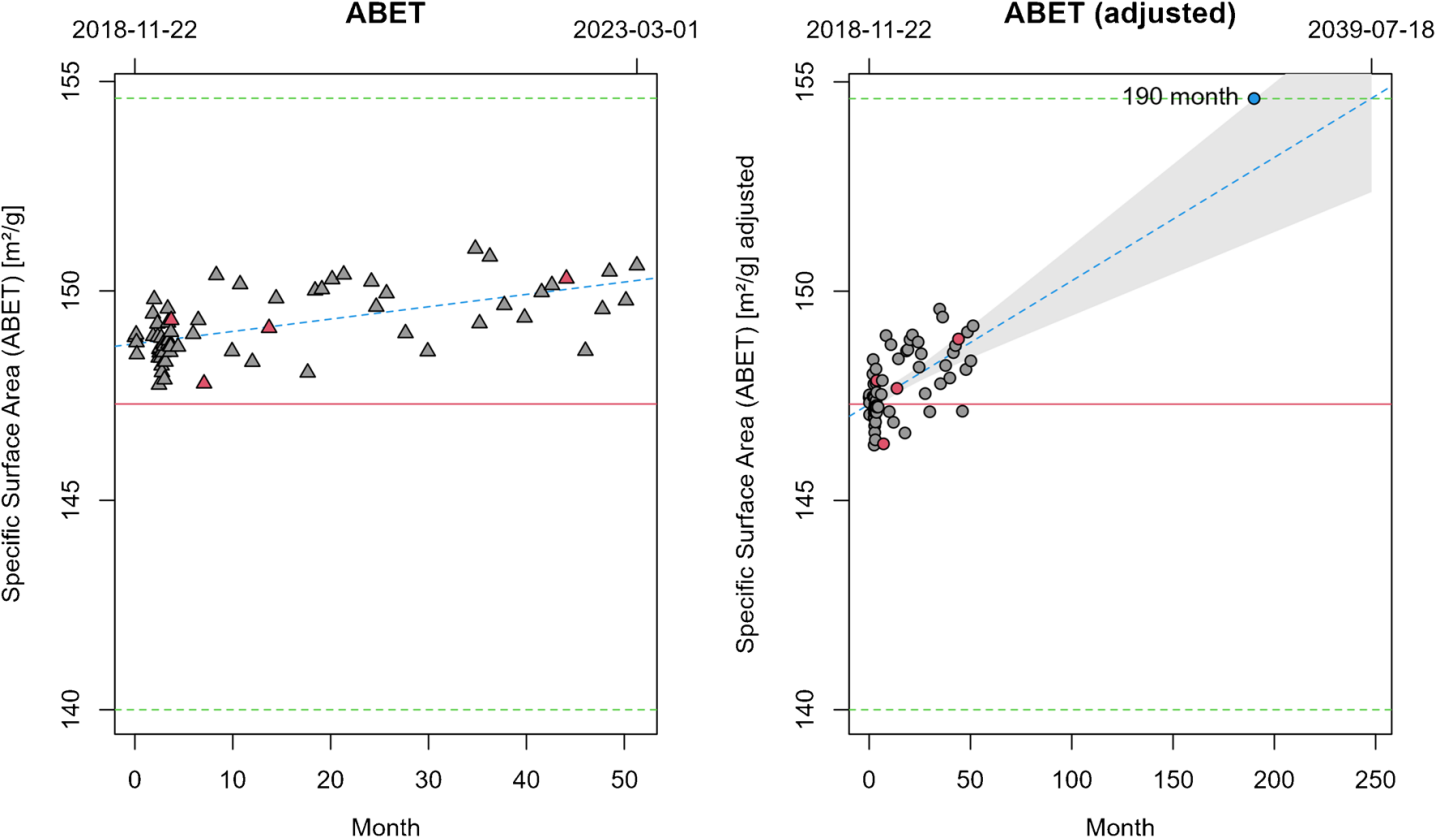
Left panel: Automatic display of the recorded monitoring data including the regression line (blue) and certified values *µ*_c_ (red line) and 2s (green lines). Right panel: Adjustment of this data with respect to *µ*_c_ and LTS calculation (prospective shelf life) in month. The gray shaded area is the 95% confidence interval of the regression line (blue). Symbols with a red background indicate comments attached to this data point

The LTS module calculates the expected lifetime of a RM (Fig. 9). Lifetime calculation is updated fully automatically with each new entry of measurement data and saves tedious input and calculations in statistical and spreadsheet software such as Excel or Origin. Specifically, the intercept *b*_0_ of the linear model is adjusted for the difference between the mean of all recorded LTS data and the mean reported as certified value on import *b*_0_′ = *b*_0_ + *μ*_LTS_ − *μ*_c_. In the following, *b*_0_′ and *b*_1_ are used to estimate the time point when the value of the certified parameter is expected to exceed the range of *μ*_c_ ± *U*. The 95% confidence interval of the regression line can be displayed on demand for more strict lifetime estimates.

The calculation results can be saved to continue the PCM process and exported as a PDF report.

### Reporting, archiving, and validation

*eCerto* allows to export most of the generated tables and figures to be included in the documentation of the CRM manufacturing report. Where standardized report templates were available at BAM (i.e., in LTS module), these were implemented in *eCerto* for user convenience. When no such standardized templates exist, *eCerto* exports the relevant results in HTML format as this facilitates maintaining a consistent layout on screen and in the printable report. It is left to the user to incorporate this data appropriately in the word processing software used for the CRM documentation.

Input data of all *eCerto* modules (H, S, and C) are stored together with the set of user options affecting the statistical output (filtered values, rounding precision, etc.) in a single RData file. This allows on the one hand a convenient way to retrospectively follow the statistical evaluation or share the analyses with collaborators, while on the other hand protecting the data against accidental manipulation which is a requirement in accredited environments.

To test for potential deviations from previous results and ensure the correct implementation of all statistical tests in *eCerto*, we reanalyzed data from the production process of several CRMs and compared the certified results. To this end, we did not observe significant deviations which is a strong indicator for a correct implementation. Version control and automatic testing were set up to ensure that *eCerto* analyses are robust and traceable also in case of future extensions of this modular software.

### Supplementary Information

Below is the link to the electronic supplementary material.

Supplementary file1 (ZIP 154 KB)

## Declarations

### Supplementary information

SI 1 Excel input of homogeneity data to reprocess data recorded during the evaluation of CRM BAM-U-115. SI 2 Excel input of stability data to reprocess data recorded during the evaluation of CRM ERM-BC715. Load this Excel file with *eCerto*, go to the Stability/Arrhenius page and enter the value -0.06276 in the input box. This value would be calculated from µ_C_ and U_abs_ which are not accessible to *eCerto* without data upload to the C module. SI 3 Excel input files of the collaborative study results to reprocess data recorded during the evaluation of CRM BAM-U-115 (provided as xlsx files numbered from 1 to 17). SI 4 RData file obtained after the evaluation of CRM BAM U-115 with *eCerto*. SI 5 RData file containing parameters Ca and Mg in the certified material ERM-ED103.
